# Post-transplant lymphoproliferative disorder involving the ovary as an initial manifestation: a case report

**DOI:** 10.1186/1752-1947-4-184

**Published:** 2010-06-18

**Authors:** Takamitsu Inoue, Shigeru Satoh, Mitsuru Saito, Yohei Horikawa, Norihiko Tsuchiya, Tomonori Habuchi

**Affiliations:** 1 Department of Urology, Akita University School of Medicine, Akita 010-8543, Japan

## Abstract

**Introduction:**

Because the normal ovary is assumed to be devoid of lymphoid tissue, it is unusual for it to be an initial manifestation of malignant lymphoma. This case is the first report, to our knowledge, of post-transplant lymphoproliferative disorder involving the ovary as an initial manifestation.

**Case presentation:**

Twenty-nine weeks after a living renal transplantation, a 38-year-old Japanese female, whose ethnic origin was Asian, presented with abdominal pain and a chronic high fever. Computed tomography revealed a right ovarian tumor and liver metastases. The patient underwent oophrectomy based on the clinical diagnosis of liver metastasis from the primary ovarian tumor. The pathological diagnosis was Epstein-Barr Virus-associated post-transplant lymphoproliferative disorder. While ovarian malignant lymphoma has a poor prognosis, complete remission of liver involvement in this case was achieved only with a reduction of immunosuppressants.

**Conclusion:**

Clinicians should remember that malignant lymphoma could initially involve the ovary, especially if the patient is immunosuppressed after transplantation therapy.

## Introduction

Post-transplant lymphoproliferative disorder (PTLD) is a type of malignant lymphoma, which is a potentially life-threatening complication that occurs in approximately 1.7% to 3.5% of solid-organ transplantation recipients [1]. While ovarian involvement of malignant lymphoma usually occurs late in the course of a disseminated disease, it is unusual to find an ovarian mass as an initial manifestation [2]. Patients with ovarian malignant lymphoma have a poor prognosis [2]. We report an unusual case of PTLD involving the right ovary as an initial manifestation, which was successfully managed with an oophrectomy followed by reduction with immunosuppressants. This case is the first report of PTLD involving the ovary as an initial manifestation.

## Case presentation

A 38-year-old Japanese female, whose ethnic origin was Asian, presented with end-stage renal failure due to hypertension from gestosis. She had been maintained with intermittent peritoneal dialysis for 10 years. Our patient underwent a living renal transplantation in January 2002 with her mother as the donor. Human leukocyte-antigen typing of the donor and recipient revealed one identical haplotype. A direct cross-match with the complement-dependent cytotoxic assay was negative. Both the recipient and donor had an immunoglobulin G (IgG) for the Epstein-Barr virus (EBV) virus-core antigen at ×320 and ×160, respectively. Pre-operative evaluation with computed tomography (CT) revealed no tumor lesions. On the third post-transplantation week, she presented with acute T-cell-mediated rejection and was treated with predonisolone (500 mg/day for two days and 250 mg for one day) and deoxyspergualin (200 mg/day for seven days). After discharge, she was administered 2000 mg/day of mycophenolate mofetil and 10 mg/day of prednisolone, and her serum tacrolimus trough level (8 ng/dL) was stable. Serum creatinine concentration was 1.1 mg/dL 28 weeks after the transplantation.

**Figure 1:**
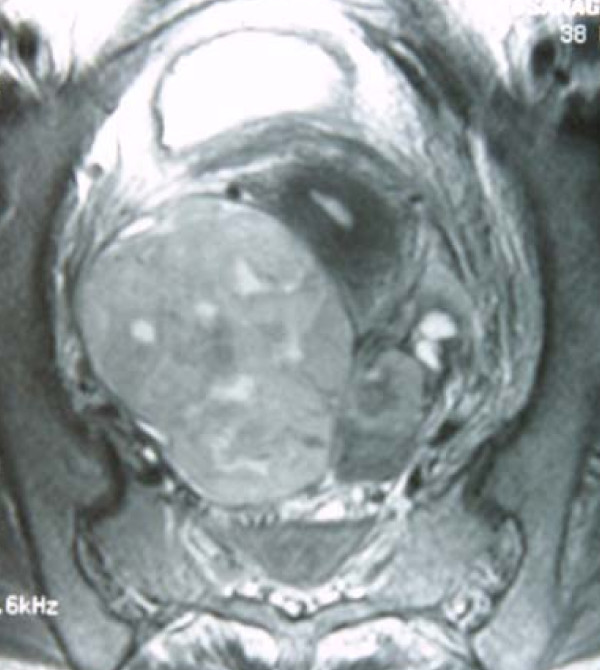
**Magnetic resonance image of the right ovarian tumor on week 27 post-transplantation**.

**Figure 2:**
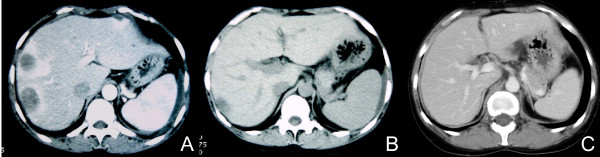
**Computed tomography before (A), four weeks after (B), and three months after (C) reduction of immunosuppression remarkably reduced that liver involvement**.

**Figure 3:**
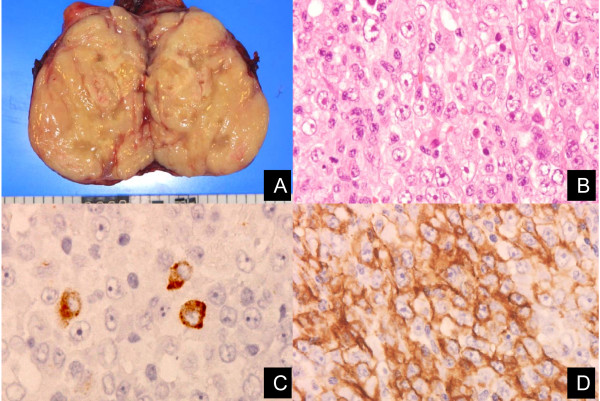
**The pathological diagnosis was Epstein-Barr virus (EBV)-associated monomorphic B-cell post-transplant lymphoproliferative disorder compatible with diffuse large B-cell lymphoma (LMP-1^+^, CD20^+^)**.

The patient was re-admitted with abdominal pain and continuous high fever 27 weeks post-transplantation. Abdominal CT and magnetic resonance imaging revealed a right primary ovarian tumor and liver metastases (Figures 1 and 2a), and her serum lactic dehydrogenase was elevated (490IU/dL). Based on the clinical diagnosis of liver metastasis from the primary ovarian tumor at the initial presentation, a right oophrectomy was performed 29 weeks post-transplantation. The pathological diagnosis was PTLD, EBV-associated monomorphic B-cell category, compatible with diffuse large B-cell lymphoma (LMP-1^+^, CD20^+^) (Figure 3).

Four weeks after the reduction of immunosuppressants with only 10 mg/day of predonisolone, CT showed remarkably reduced liver involvement (> 95%) (Figure 2b) and the serum lactic dehydrogenase concentration decreased to the normal range (117IU/dL). Three months after the reduction of immunosuppressants, a CT indicated complete remission of the liver involvement (Figure 2c). The serum creatinine concentration was maintained at 1.1 mg/dL, and no evidence of the disease was revealed 68 months post-transplantation. The patient had been administered only 10 mg/day of predonisolone during that period.

## Discussion

Malignant lymphomas involving the ovaries as the final condition of disease occur at a frequency of 26% at necropsy or autopsy [3]. However, less than 1% of patients with malignant lymphoma initially present with enlarged ovaries [2]. In a previous report of approximately 9500 women with lymphomas, only 19 (0.2%) were known to have an initial manifestation in the ovary [3]. The infrequency of primary ovarian lymphoma was assumed to be due to the lack of lymphoid tissue in the normal ovary; however, recent studies have found benign lymphoid aggregates in approximately half of normal ovaries [2]. Our patient was initially diagnosed with liver metastasis from primary ovarian cancer, and she underwent oophrectomy based on the knowledge that initial ovarian involvement with PTLD is infrequent.

Malignant lymphoma that initially presents with enlarged ovaries is categorized into primary and secondary ovarian lymphomas. True primary ovarian lymphoma is considered curable only by oophrectomy and carries a favorable prognosis, whereas patients with the secondary disease have a poor prognosis [2]. Fox *et al*. proposed the stringent criteria for the diagnosis of primary ovarian lymphoma; the lymphoma is clinically confined to the ovary at the time of diagnosis, and a full investigation fails to reveal evidence of lymphoma elsewhere [4]. Our case of PTLD that involved the right ovary and liver was not applicable to this criterion. The limited focus to the liver was one of the reasons for the favorable prognosis in this case.

PTLD is one of the most worrisome complications after organ transplantation. The incidence rate of PTLD is 1.7% to 3.5% or 33.27/10,000 person-years of solid-organ transplant recipients [1,5,6]. Pre-transplant recipient EBV seronegativity is a well-established risk factor for developing PTLD [7]. Immortal B-cells infected with EBV proliferate indefinitely in immunosuppressed patients, whereas EBV-specific cytotoxic T-cells (CD8^+^) destroy infected B-cells in healthy humans. In countries where Burkitt's lymphoma due to EBV infection is endemic, ovarian involvement is the second most common form of Burkitt's lymphoma after jaw involvement [8].

Reducing immunosuppressants is the first-choice therapy for PTLD. In a previous report, 47% of PTLD patients had a complete remission with only reduction of immunosuppressants and 58% responded with reduction of immunosuppressants and concurrent surgical resection [1]. However, Tsai *et al*. remarked that patients with multiple poor prognostic factors, such as elevated lactate dehydrogenase ratio, significant organ dysfunction, or multi-organ PTLD should be considered for other therapies in combination with the reduction of immunosuppressants [1]. Our patient had an elevated lactate dehydrogenase ratio and multi-organ PTLD. Recently, anti-CD20 monoclonal antibody (rituximab) monotherapy or a combination therapy with combination chemotherapy with cyclophosphamide, hydroxydaunorubicin, vincristine, and prednisolone (CHOP) was reported to be effective for PTLD [9,10]. These alternative therapies would have been considered if the reduction of immunosuppressants was not effective in this case.

## Conclusion

Our patient was initially diagnosed with a liver metastasis from primary ovarian cancer and underwent oophrectomy based on the knowledge that initial ovarian involvement with PTLD is infrequent. This case is the first report of PTLD involving the ovary as an initial manifestation. A complete remission from the liver involvement was achieved only with reduction of immunosuppressants. Clinicians should remember that malignant lymphoma could initially involve the ovary, especially if the patient is immunosuppressed after transplantation therapy.

## Abbreviations

CHOP: combination chemotherapy with cyclophosphamide, hydroxydaunorubicin, vincristine, and prednisolone; CT: computed tomography; EBV: Epstein-Barr virus; LDH: lactate dehydrogenase; PTLD: post-transplant lymphoproliferative disorder.

## Consent

Written informed consent was obtained from the patient to publish this case report and any accompanying images. A copy of the written consent is available for review by the Editor-in-Chief of this journal.

## Competing interests

The authors declare that they have no competing interests.

## Authors' contributions

TI contributed to the management of the patient, writing of the manuscript, and the literature review. SS, MS, and YH contributed to the management of the patient as physicians in charge. NT and TH advised on treatment policy. All authors have read and approved the final manuscript.
